# Radiofluorination of a Pre-formed Gallium(III) Aza-macrocyclic Complex: Towards Next-Generation Positron Emission Tomography (PET) Imaging Agents

**DOI:** 10.1002/chem.201405812

**Published:** 2015-02-04

**Authors:** Rajiv Bhalla, William Levason, Sajinder K Luthra, Graeme McRobbie, George Sanderson, Gillian Reid

**Affiliations:** [a] Centre for Advanced Imaging, University of Queensland BrisbaneQueensland 4072 (Australia); [b] Chemistry Department, University of SouthamptonHighfield, Southampton, SO17 1BJ (UK); [c] GE Healthcare UKWhite Lion Road, Amersham, HP 7 9LL (UK)

**Keywords:** imaging agents, gallium, positron emission tomography, radiofluorination

## Abstract

As part of a study to investigate the factors influencing the development of new, more effective metal-complex-based positron emission tomography (PET) imaging agents, the distorted octahedral complex, [GaCl(L)]⋅2 H_2_O has been prepared by reaction of 1-benzyl-1,4,7-triazacyclononane-4,7-dicarboxylic acid hydrochloride (H_2_L⋅HCl) with Ga(NO_3_)_3_⋅9 H_2_O, which is a convenient source of Ga^III^ for reactions in water. Spectroscopic and crystallographic data for [GaCl(L)]⋅2 H_2_O are described, together with the crystal structure of [GaCl(L)]⋅MeCN. Fluorination of this complex by Cl^−^/F^−^ exchange was achieved in high yield by treatment with KF in water at room temperature over 90 minutes, although the reaction was complete in approximately 30 minutes if heated to 80 °C, giving [GaF(L)]⋅2 H_2_O in good yield. The same complex was obtained by hydrothermal synthesis from GaF_3_⋅3 H_2_O and Li_2_L, and has been characterised by single-crystal X-ray analysis, IR, ^1^H and ^19^F{^1^H} NMR spectroscopy and ESI^+^ MS.

Radiofluorination of the pre-formed [GaCl(L)]⋅2 H_2_O has been demonstrated on a 210 nanomolar scale in aqueous NaOAc at pH 4 by using carrier-free ^18^F^−^, leading to 60–70 % ^18^F-incorporation after heating to 80 °C for 30 minutes. The resulting radioproduct was purified easily by using a solid-phase extraction (SPE) cartridge, leading to 98–99 % radiochemical purity. The [Ga^18^F(L)] is stable for at least 90 minutes in 10 % EtOH/NaOAc solution at pH 6, but defluorinates over this time scale at pH of approximately 7.5 in phosphate buffered saline (PBS) or human serum albumin (HSA). The subtle role of the Group 13 metal ion and co-ligand donor set in influencing the pH dependence of this system is discussed in the context of developing potential new imaging agents for PET.

## Introduction

Fluorine-18, a positron-emitting isotope with a half-life of 109.8 minutes, is readily produced using a cyclotron and has become the radioisotope of choice for many medical imaging applications. The use of metal chelate-based complexes as a route towards new types of radioimaging agents for positron emission tomography (PET) by using ^18^F offers an alternative strategy towards new PET agents from the widely studied organo-fluorine-based agents. A consequence of the relatively short half-life, *t*_1/2_, of ^18^F is that for medical applications rapid, late-stage radiolabelling is particularly desirable, ideally this should be the final step of the synthesis. The ability to introduce the radiolabel in water is also attractive in simplifying the procedure.

In addition to very elegant recent work towards organo-fluorine-based agents,[1] there has been a surge of research activity targeted for developing new inorganic ^18^F agents, including those centred upon B=F,[2] Si=F systems[3] and also metal coordination complexes, based on Al=F and Ga=F species.[4–6] The strength of the fluorine-element bond being formed during the radiofluorination is one of several key parameters in determining the suitability of a particular agent. Within the metal-chelate-based systems, formation of a strong M=F bond on a labile metal ion can allow reactions to proceed quickly and under relatively mild conditions. The strength of the other metal-co-ligand interactions are also important depending upon the mechanism that prevails for the introduction of the F^−^, and also for the stability of the final metal–fluoride complex under physiological conditions. The trivalent Group 13 metal ions, Al, Ga, In, are redox inactive, have relatively low toxicities and have well-defined coordination numbers for particular ligand sets.[7] These are important considerations in simplifying the solution chemistry of potential imaging agents in vivo. Other important factors include the nature of the chelating ligand to provide stability and scope for functionalisation to allow conjugation to the relevant biomolecules. Macrocyclic ligands offer advantages, because they tend to form very robust complexes with metal ions, which are often resistant to demetallation.

**Scheme 1:**
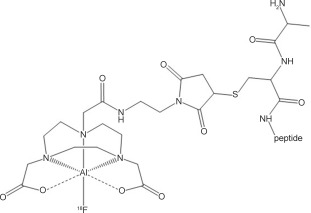


The work of McBride and co-workers[4] has demonstrated that careful choice of ligand type and metal allows easy access to ^18^F-containing compounds founded upon Al^III^-triaza-macrocyclic complexes (Scheme 1). Recently Wan and co-workers have reported that this “Al–^18^F” system has been translated into the clinic, by the simple addition of carrier-free [^18^F] fluoride to a “pre-formed kit” containing an RGD NOTA (RGD=arginine-glycine-aspartic acid; NOTA=1,4,7-triazacyclononane-1,4,7-triacetic acid) conjugate and AlCl_3_⋅6 H_2_O buffered to pH 4, and heating to 100 °C.[8] The success of McBride’s approach has stimulated efforts to develop the Al chemistry further to create new generation agents by using a coordination chemistry approach.[5]

We recently reported an alternative route towards stable “Ga–^18^F” labelled compounds by using a pre-formed chloride-containing Ga^III^ precursor complex that undergoes rapid halide (Cl/F) exchange within the metal coordination sphere under mild conditions, that is, at room temperature in weakly acidic aqueous MeCN solution.[6] The resulting [Ga^18/19^F_3_(BzMe_2_-tacn)] (BzMe_2_tacn=1-benzyl-4,7-dimethyl-1,4,7-triazacyclononane), containing the neutral tridentate tacn macrocycle, showed very good stability in phosphate buffered saline (PBS) at pH 7.5 for at least two hours. As well as demonstrating the viability of this approach by using pre-formed Group 13 metal complexes, this study also revealed some important differences in the chemistry and properties of the corresponding Al^III^, Ga^III^ and In^III^ systems.

To develop this approach towards new PET imaging agents, there is considerable scope for exploring the underlying chemistry and for optimising the metal–ligand system. An optimal target for clinical use is a pre-formed complex (agent), provided in kit form, that undergoes rapid ^18^F incorporation under mild conditions and at nanomolar concentration. This should ideally be a single synthetic step in the clinic that requires no purification post labelling, or, if purification is necessary, in which a simple cartridge-based method can be applied.

**Scheme 2:**
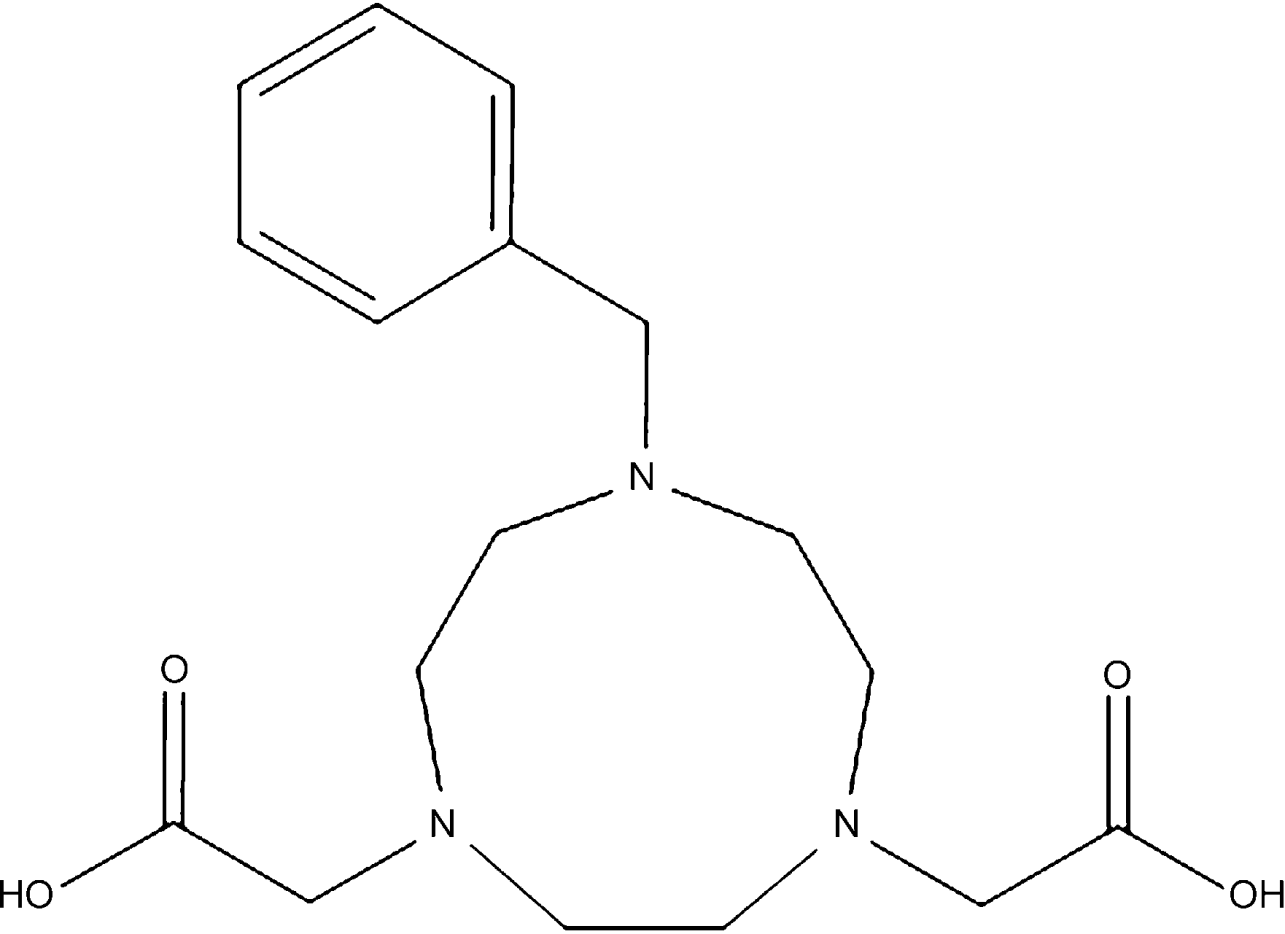


Herein, we demonstrate the successful radiofluorine-labelling of a pre-formed gallium chloride chelate complex based on the anionic pendant arm azamacrocyclic ligand, 1-benzyl-1,4,7-triazacyclononane-4,7-dicarboxylate (L^2−^, the dianion of H_2_L in Scheme 2) on a nanomolar scale in water by using carrier-free ^18^F^−^, in which the resulting radiocomplex has been purified by using a solid-phase extraction (SPE) cartridge.

## Results and Discussion

### Preparative scale synthesis of [GaX(L)] (X=Cl or F)

The success of the room temperature halide-exchange reaction of the pre-formed [MCl_3_(R_3_-tacn)] complexes in aqueous solution[6] confirms that the Ga=F bonds are sufficiently stable to be considered for imaging applications and prompted further work to determine whether the same general method could be employed with the dicarboxylate pendant-arm ligand, 1-benzyl-1,4,7-triazacyclononane-4,7-dicarboxylate (tacn; L^2−^). At first sight, this ligand may offer some advantages over the neutral BzMe_2_-tacn; it is potentially pentadentate with an N_3_O_2_ donor set, leaving only the one coordination site required for ^18^F^−^ incorporation, while also contributing to the stability of the metal complex through a combination of both the macrocyclic and chelate effects. This moiety may also allow ^18^F to be incorporated without the need for any ^19^F and provides a direct comparison with the work of Jeong and co-workers.[5a] The ligand has been synthesised as both the dilithium salt (Li_2_L)[9] and as the carboxylic acid (H_2_L⋅HCl).[5a]

Preparation of the precursor complex [GaCl(L)] was initially undertaken through reaction of GaCl_3_ in anhydrous MeCN with a solution of Li_2_L in dry MeOH, giving a yellow orange crude solid that was poorly soluble in common solvents (H_2_O, MeOH, MeCN). Mass spectrometry, IR and ^1^H NMR spectroscopic data are consistent with the target complex, indicating pentadentate coordination of the macrocyclic ligand, with a single Cl^−^ ligand completing the distorted octahedral coordination environment at Ga^III^. However, purification and separation of the inorganic by-product (LiCl) proved to be challenging. Furthermore, the use of GaCl_3_, which is both very readily hydrolysed and reactive, as the source of the Ga=Cl unit in the product was undesirable considering the goal to be able to create and radiolabel sub one milligram quantities of the pre-formed complex for imaging.

Therefore, we sought an alternative source of Ga^III^, which would be better suited for use in aqueous solution. The compound Ga(NO_3_)_3_⋅*x* H_2_O is commercially available (Aldrich), and has been identified as the nona-hydrate in the solid state.[10] Reaction of Ga(NO_3_)_3_⋅9 H_2_O with one molar equivalent of H_2_L⋅HCl on a preparative scale in aqueous solution gave a yellow solid after work-up. Spectroscopic data are consistent with the formulation [GaCl(L)]. The solid was recrystallised from MeCN/Et_2_O to remove impurities. The chelation reaction is slow at room temperature in concentrated solution (ca. 12 h), but proceeds significantly faster at 85 °C. The reaction may also be performed upon heating at reflux in MeOH from which the desired product precipitates as a pale yellow solid. It is stable for many months in the solid state, and the ^1^H NMR spectrum (D_2_O) is unchanged after four weeks in solution. The [GaCl(L)] is soluble in H_2_O and MeCN, and is poorly soluble in chlorocarbons.

The ^1^H NMR spectrum (in D_2_O) of the [GaCl(L)] is significantly shifted and more complex than that of the H_2_L⋅HCl. The CH_2_ protons of the benzyl group are split into an AB quartet (^2^*J*_HH_ 13.7 Hz) indicating that the tacn-dicarboxylate ligand is locked by N_3_O_2_ coordination, leading to diastereotopic inequivalence of the CH_2_ protons in the carboxylate groups. The tacn protons also appear as second-order multiplets. ESI^+^ mass spectrometry gave *m*/*z* 402.1 (100 %), with an associated ^69/71^Ga isotope pattern, corresponding to the monocation, [Ga(L)]^+^. The IR spectrum (Nujol mull) showed the expected carboxylate CO bands of the ligand were shifted to low frequency by approximately 50 cm^−1^ compared with H_2_L⋅HCl itself. The IR spectrum also showed the expected Ga=Cl stretching vibration (375 cm^−1^) and evidence for H-bonded water (*ν*(OH)=3750, *δ*(HOH)=1648 cm^−1^). On the basis of these data, we concluded that the NO_3_^−^ groups from the Ga(NO_3_)_3_⋅9 H_2_O precursor are not retained in the product, being replaced by the two carboxylate pendant arms of the macrocycle and one chloride anion, the latter derived from the H_2_L⋅HCl, in the distorted octahedral complex.

Confirmation of this formulation came from a structure determination on a very weakly diffracting crystal obtained by slow evaporation from an MeCN solution of [GaCl(L)].

**Figure 1:**
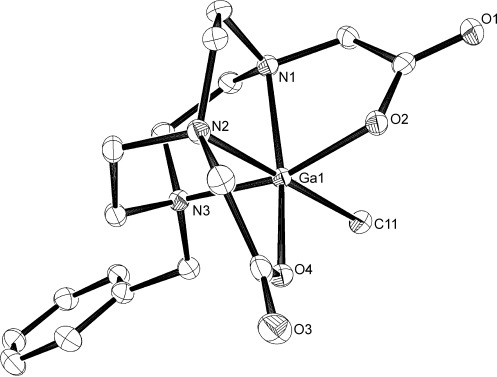
Crystal structure of [GaCl(L)]⋅MeCN with atom numbering scheme. Hydrogen atoms and lattice MeCN are omitted for clarity. Thermal ellipsoids are drawn at 50 % probability level. Selected bond lengths [Å]: Ga1=Cl1 2.2792(5), Ga1=O4 1.9139(12), Ga1=O2 1.9824(13), Ga1=N1 2.0970(15), Ga1=N2 2.1222(15), Ga1=N3 2.1243(15).

The structure of [GaCl(L)]⋅2 H_2_O shows a distorted octahedral coordination environment at gallium through pentadentate coordination of L^2−^ by its N_3_O_2_ donor set and one chloride ligand (Figure S1 a in the Supporting Information). The complex crystallises as a dihydrate, with one of the lattice H_2_O molecules H bonded to the O atoms of the carboxylate CO groups (Figure S1 b in the Supporting Information). This contrasts with the F⋅⋅⋅H=OH interactions observed in the [MF_3_(R_3_-tacn)] (M=Al, Ga, In).[6] The quality of the [GaCl(L)]⋅2 H_2_O crystals was poor, characterised by weak reflection data. However, recrystallization from MeCN solution over several weeks gave crystals of [GaCl(L)]⋅MeCN of much higher quality. The Ga chelate complex (Figure 1) is essentially identical to that in the dihydrate, with *d*(Ga=Cl)=2.2793(5) Å (*d*=distance), although the lattice MeCN does not interact with the Ga species.

Before the Cl/F halide exchange reaction was attempted by using the [GaCl(L)], we sought a method to obtain the corresponding Ga=F complex as a model compound to provide a well-defined spectroscopic fingerprint in preparation for the exchange studies. Given the success of hydrothermal synthesis in our previous work,[6] a similar method was employed. Reaction of GaF_3_⋅3 H_2_O with Li_2_L (to ensure exclusion of Cl^−^) under hydrothermal conditions resulted in the formation of the complex [GaF(L)]⋅2 H_2_O as a yellow solid after work-up. The precipitation of LiF provides a driving force towards the complexation. The ^1^H NMR spectrum of the product showed a complex pattern consistent with ligand coordination. The pattern showed small chemical-shift differences from the chloride analogue, [GaCl(L)], and significant differences from the spectrum of the Li_2_L. The ^19^F{^1^H} NMR spectrum revealed a single resonance at *δ*=−184.2 ppm, consistent with a Ga=F containing compound.[6] The ESI^+^ mass spectrum of the compounds showed *m*/*z* 402.1 (100 %), with the expected isotope pattern for [Ga(L)]^+^. The IR spectrum showed a single, broad Ga=F stretching band ($\tilde \nu $
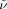
=568 cm^−1^). Evidence for H-bonded water was also evident from the IR spectrum. Slow evaporation of water from the hydrothermal reaction solution gave small crystals suitable for single crystal X-ray diffraction.

**Figure 2:**
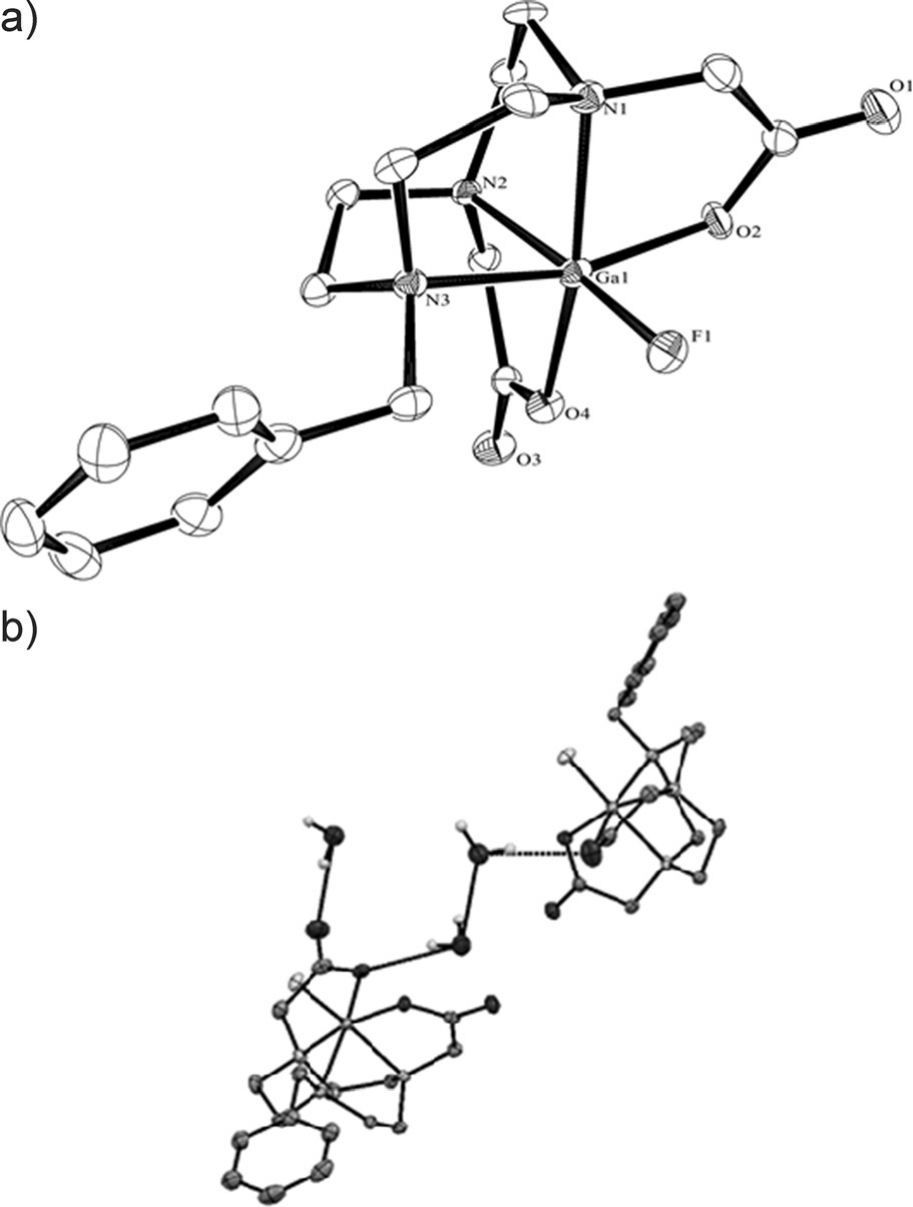
a) ORTEP representation of [GaF(L)]⋅2 H_2_O. Thermal ellipsoids are drawn at 50 % probability, hydrogen atoms (except those associated with the lattice H_2_O molecules) are omitted for clarity. Selected bond lengths [Å]: Ga1=F1 1.821(2), Ga1=O4 1.940(3), Ga1=O2 1.980(3), Ga1=N1 2.082(4), Ga1=N2 2.111(3), Ga1=N3 2.146(3). b) Diagram showing a portion of the extended structure of [GaF(L)]⋅2H_2_O (Ga pink; F green; O red; N blue; C grey).

X-Ray structural characterisation confirmed the expected distorted octahedral coordination at gallium, through a pentadentate L^2−^ ligand and one terminal F^−^ ligand (Figure 2 [a]). The Ga=F bond length was found to be 1.821(2) Å, one of the shortest Ga=F bonds observed crystallographically, and even slightly shorter than *d*(Ga=F) in [GaF_3_(Me_3_-tacn)]⋅4 H_2_O (1.851(3), 1.858(3), 1.881(3) Å),[6] although the latter exhibited extensive F⋅⋅⋅H=OH hydrogen bonding to lattice water, and these may be responsible for longer Ga=F bond lengths in this complex.[11,12]

Comparison with the analogous Al=F complex, [AlF(L)]⋅2 H_2_O[5a] showed *d*(Al=F)=1.7090(14) Å, leading to a difference in *d*(M=F) of 0.11 Å. This is larger than would be expected based on the difference in covalent radii from Al^3+^ to Ga^3+^.[13] Similarly, the difference between *d*(Al=O) and *d*(Ga=O) is approximately 0.10 Å. Comparison of the M=N bond strengths showed that *d*(Ga=N) lies in the range from 2.082(4) to 2.146(3) Å, whereas *d*(Al=N) lies between 2.0497(18) and 2.1125(18) Å, that is, there is a smaller effect on the M=N distances between the aluminium and gallium complexes. The presence of the macrocyclic ring may also play a role here.

Similar to the chloride analogue (ESI), the [GaF(L)] complex crystallises as a dihydrate; although they are isostructural, they are not isomorphous due to differences in the H-bonding arrangement. In the fluoride complex, both lattice waters are involved in H bonding (Figure 2 [b]).

The fluoride complex is stable in the solid state, and for several weeks in aqueous solution (in which the pH was measured to be ca. 4) and in other acidic media. Addition of aqueous KOH to an aqueous solution of the [GaF(L)] complex to bring the solution to pH 7 led to release of F^−^ within 5–10 minutes. This is supported by the loss of the ^19^F{^1^H} NMR resonance observed for the complex and the growth of a resonance due to F^−^ (*δ*=−123.7 ppm).

### Cl^−^/F^−^ Exchange

Reaction of [GaCl(L)]⋅2 H_2_O in aqueous MeCN (unbuffered) with one molar equivalent of aqueous KF leads to complete conversion to the corresponding fluoride complex. The exchange proceeds to completeness at a moderate rate (ca. 3 h) at room temperature, but is significantly accelerated (requiring ca. 45 min) if heated to 80 °C. Spectroscopic analysis of the resulting product matched to that observed for [GaF(L)] synthesised hydrothermally. The Cl/F halide exchange may also be performed in buffered NaOAc solution (pH 4).

### ^18^F Radiolabelling

**Figure 3:**
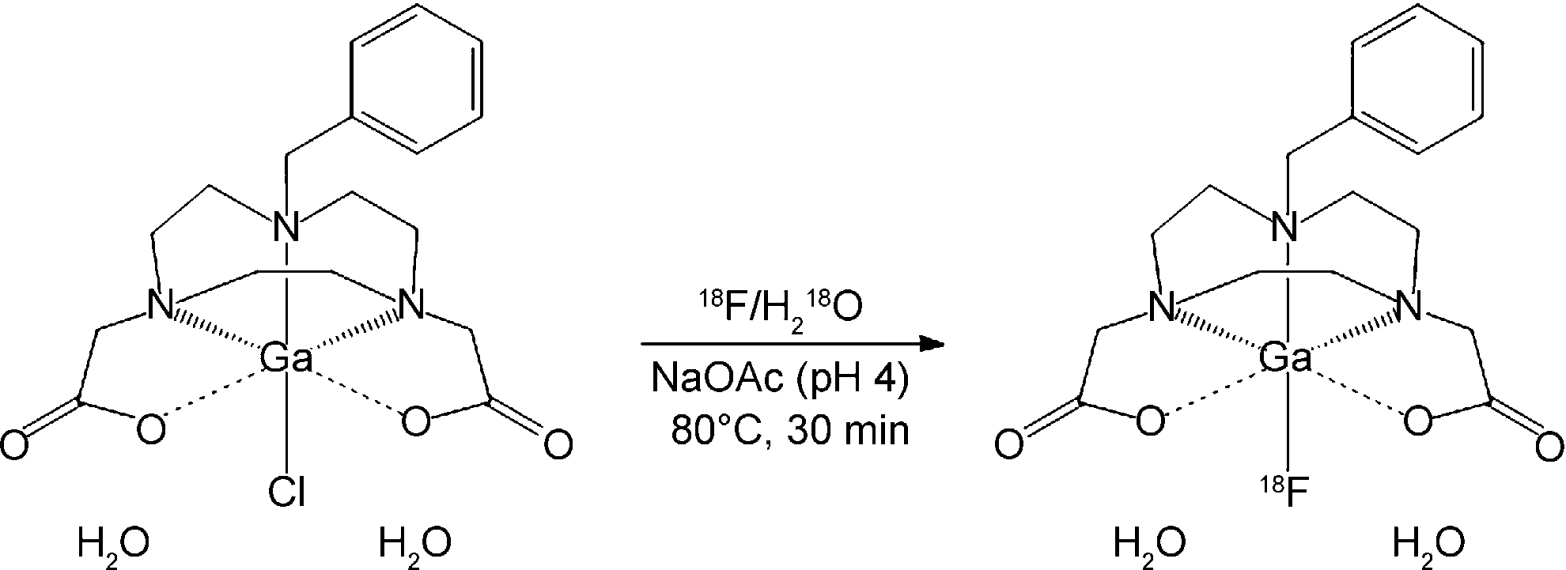
Radiofluorination conditions for preparation of [Ga^18^F(L)].

Having demonstrated the ability to successfully perform halide exchange on the pre-formed [GaCl(L)] complex on a preparative scale by using ^19^F^−^, radiofluorination was attempted (Figure 3). Carrier-free ^18^F/^18^OH_2_ (500–1000 MBq) was added to 0.1 mg of the chloride precursor (210 nmol) dissolved in NaOAc buffer (pH 4) and left to react at room temperature for 30 minutes. HPLC analysis of the crude reaction mixture showed a radiopeak at *R*_t_ 6–6.2 minutes corresponding to [Ga^18^F(L)] integration of the radiopeak indicated approximately 30 % incorporation of ^18^F into the gallium complex at room temperature after 30 minutes (Figure S2 in the Supporting Information). Heating the reaction solution to 80 °C for 30 minutes led to a significant increase in the incorporation to 65–70 % (Figure S3 in the Supporting Information).

A small sample of the crude product (*R*_t_=6 min) was removed and injected directly onto an ESI^+^ mass spectrometer. Similar to the model compound, the parent ion was not observed; however, peaks attributed to the monocation, [GaL]^+^ (*m*/*z*=402.1) and [{GaF(L)}_2_+H_3_O]^+^ (*m*/*z*=863.2) were observed (see the Supporting Information, Figures S4–S7). Together with the HPLC radiotrace, this confirmed the formation of the target Ga=F complex, [Ga^18^F(L)].

The crude ^18^F-labelled compound was then purified by trapping it on an hydrophilic lipophilic branched (HLB) cartridge and eluted with EtOH/H_2_O. This purification process is very efficient, giving radioactive concentrations (RACs) of up to 100 MBq mL^−1^. The purified radiochemical product is stable for at least 180 minutes when formulated in 10 % EtOH/NaOAc at pH 6 (Figure S8 a–c in the Supporting Information). However, when formulated in 10 % EtOH/PBS (pH 7.5), the product is unstable, with the initial radiochemical purity (RCP) of 95—98 %, decreasing to 40 % after 20 minutes and 2 % after 90 minutes (Figure S9 a–c in the Supporting Information). This is consistent with our observation that the non-radioactive [GaF(L)] complex is unstable in aqueous KOH at pH 7. Compound [Ga^18^F(L)] also liberates ^18^F^−^ when formulated into human serum albumin (HSA; pH 7.4; Figure S10 a–c in the Supporting Information).

These data suggest that the stability is strongly pH dependant (Table S2 in the Supporting Information), although the presence of competing ions in the PBS and HSA formulations may also play a role.

The trend in stability with pH can be replicated on a preparative scale when K^19^F was used as the fluorinating agent. The fluoride liberated at pH 7.5 (in PBS) was readily observed by ^19^F{^1^H} NMR spectroscopy. It is notable that the [GaF_3_(BzMe_2_-tacn)] is stable for several hours (with no evidence of defluorination) in PBS,[6] both on a tracer scale concentration (micromolar) and on a preparative scale (mmol), and hence, it is clear that neither the Ga=F bonds nor the Ga=N(tacn) bonds are inherently unstable at pH 7.5. Therefore, it is likely that the pH-dependent instability of [GaF(L)] is associated primarily with the Ga=O(carboxylate) bonds, with subsequent loss of F^−^. It is known from previous work on F^−^/H_2_O exchange on Al^III^ complexes[14] that small changes in the steric environment can change the substitution mechanism (e.g., from D to I), hence, the steric and/or electronic changes at Ga^III^ caused by the different donor sets in [GaF(L)] compared with [GaF_3_(R_3_-tacn)] may be the basis for the observed instability of the former at pH 7.5. Because Ga^III^ is less Lewis acidic than Al^III^,[12,15] the introduction of the Ga=O (carboxylate) bonds in [GaF(L)] may lead to initial cleavage of the Ga=O bond at pH 7.5 (e.g., by H_2_O or an anion present in the formulation), destabilising the Ga^III^ coordination sphere to cause the observed decomposition. Hence, the experimental observations from this study suggest that modifications to the macrocyclic pendant groups either, for example, by increasing the steric bulk at the carboxylate functions, or by changing the carboxylate functions to other anionic donor groups could be important, leading to improved stability of M=^18^F complexes.

The [Ga^18^F(L)] may also be synthesised by a one-pot method. Reaction of Ga(NO_3_)_3_⋅9 H_2_O and Li_2_L (1:1) in NaOAc (pH 4) with ^18^F/^18^OH_2_ resulted in up to 80 % incorporation of ^18^F into the gallium complex after heating at 80 °C for 30 minutes. This product may also be purified by SPE, as was described above. The purified product showed similar defluorination in 10 % EtOH/PBS over 90 minutes at pH 7.4.

## Conclusion

This work describes a method for the preparation of [GaCl(L)]⋅2 H_2_O, containing Ga^III^ in a distorted octahedral environment provided by the pentadentate L^2−^ ligand and one Cl^−^ (derived from H_2_L⋅HCl), using the commercially available and easy-to-handle Ga(NO_3_)_3_⋅9 H_2_O as a convenient source of Ga^III^ in water. The corresponding fluoride complex, [GaF(L)]⋅2 H_2_O, has been synthesised by a hydrothermal route to provide crystallographic and spectroscopic data, and also on a bulk scale by Cl/F exchange from [GaCl(L)]⋅2 H_2_O with K^19^F in (unbuffered) water, as a model for the radiofluorination reaction.

Direct radiofluorination at nanomolar concentration by treatment of this pre-formed [GaCl(L)]⋅2 H_2_O with carrier-free ^18^F^−^ in water at pH 4 (NaOAc) at room temperature has also been demonstrated, while heating to 80 °C for 30 minutes increases the yield of [Ga^18^F(L)] giving 65–70 % ^18^F incorporation. The crude product was readily purified using an SPE cartridge, and showed excellent radiochemical stability at pH 6 (10 % EtOH/NaOAc). Defluorination was observed when [Ga^18^F(L)] was formulated in PBS and HSA (pH 7.5 and 7.4, respectively). The instability of [GaF(L)] at high pH is attributed primarily to the presence of the coordinated carboxylate groups in L^2−^. This also demonstrates subtle, but important differences in the behaviour of Ga^III^ versus Al^III^ with this dicarboxylate co-ligand; the higher Lewis acidity of Al^III^ is more manifested towards the anionic ligand groups in [AlF(L)]. This is in accord with structural data, which showed that the M=O and M=F bond lengths are longer (by ca. 0.1 Å) for Ga over Al, whereas the M=N bond lengths are more similar. The significance of the precise metal–co-ligand coordination on the stability of the radiofluoride Ga^III^ complex with pH demonstrated in this work suggests that careful tuning of the steric and electronic properties of the anionic pendant groups on the tacn should allow optimisation of the design of new improved metal-complex-based imaging agents.

## Experimental Section

Reactions were performed in standard lab glassware when appropriate. Water was freshly distilled before use. All other solvents used were of HPLC grade quality. ESI mass spectrometry was performed by using a Waters (Manchester, UK) ZMD mass spectrometer equipped with a single quadrupole analyser. Samples were introduced to the mass spectrometer by flow injection using a Waters 600 pump (flow rate 0.1 mL min^−1^ MeCN) and Waters 2700 autosampler. ^1^H and ^19^F{^1^H} NMR spectra were recorded in solution in deuterated H_2_O or methanol on a Bruker DPX-400 or AV-400 spectrometers and are referenced to the residual solvent protons (^1^H) and CCl_3_F (^19^F) at 298 K. IR spectra were recorded neat (oils) or as Nujol mulls (solids) between CsI plates by using a Perkin–Elmer Spectrum 100 spectrometer over the range 4000–200 cm^−1^. Microanalyses were undertaken by Stephen Boyer at London Metropolitan University. Compounds Bz(CH_2_CO_2_H)_2_-tacn⋅HCl (H_2_L⋅HCl)[5a] and Li_2_[Bz(CH_2_CO_2_)_2_-tacn] (Li_2_L)[9] were prepared by using the literature methods; Ga(NO_3_)_3_⋅9 H_2_O and GaF_3_⋅3 H_2_O were obtained from Aldrich and used as received.

### Synthesis of [GaCl(L)]⋅2 H_2_O

A solution of H_2_L⋅HCl (0.111 g, 0.332 mmol) in freshly distilled H_2_O (3 mL) was added dropwise to a solution of Ga(NO_3_)_3_⋅9 H_2_O (0.139 g, 0.332 mmol) in H_2_O (3 mL). The yellow solution was heated to 85 °C for 2 h. The solution was then cooled to RT, and the volatiles were removed under high vacuum upon gentle heating (ca. 40 °C). The resulting yellow solid was washed with MeCN, and the solution was filtered to remove undissolved particulates, before concentrating this to approximately 50 % volume in vacuo. Treatment of the solution with diethyl ether led to precipitation of a yellow orange solid, which was isolated by filtration and dried under high vacuum. Yield: 0.032 g, 21 % (orange solid.). ^1^H NMR (400 MHz, D_2_O, 298 K): *δ*=2.99–2.81 (m, [2 H], tacn CH_2_), 3.38–3.10 (m, [6 H], tacn CH_2_), 3.49–3.42 (m, [2 H], tacn CH_2_), 3.69–3.63 (m, [2 H], tacn CH_2_), 3.76–3.74 (d, [2 H], ^2^*J*_HH_ 10.5 Hz, N-C*H*_2_CO_2_), 3.82 (br s, [2 H], N=C*H*_2_CO_2_), 4.06–4.02 (d, [1 H], ^2^*J*_HH_ 13.7 Hz, Bz CH_2_), 4.34–4.31 (d, [1 H], ^2^*J*_HH_ 13.8 Hz, BzCH_2_), 7.48 ppm (s, [5 H], ArCH); ^13^C{^1^H} NMR (400 MHz, D_2_O, 298 K): *δ*=45.8, 51.5, 51.8, 52.4, 52.7, 53.4, 60.4, 62.0, 128.8, 132.2, 149.2, 175.4 ppm; ESI^+^ (MeCN): *m*/*z* 402.1 (100 %) [GaL]^+^; IR (Nujol): $\tilde \nu $
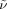
=3619 (H_2_O), 1749, 1641 (CO), 376 cm^−1^ (Ga=Cl); elemental analysis calcd for C_17_H_27_ClGaN_3_O_6_ (473.08): C 43.0, H 5.7, N 8.9; found: C 42.8, H 5.5, N 8.8 %. Weakly diffracting crystals were grown upon slow evaporation of a MeCN solution of the complex.

### Synthesis of [GaF(L)]⋅2 H_2_O

*Method 1:* GaF_3_⋅3 H_2_O (0.052 g, 0.288 mmol) and Li_2_L (0.100 g, 0.288 mmol) were added to freshly distilled H_2_O (7 mL). The mixture was transferred into a Teflon cup. The cup was placed into a stainless steel pressure vessel. The reaction was heated to 180 °C for 18 h. The vessel was cooled gradually to RT. The grey LiF by-product was removed by filtration, leaving a yellow solution. Removal of the volatiles in vacuo gave a light brown solid, which was washed with *n*-hexane and dried under high vacuum. Yield: 0.072 g, 54 % (brown solid). ^1^H NMR (300 MHz, CD_3_OD, 298 K): *δ*=2.66 (m, [2 H], tacn CH_2_), 2.73 (s, [2 H], tacn CH_2_), 2.84 (br s, [2 H], tacn CH_2_), 3.15–2.99 (br m, [6 H], tacn CH_2_), 3.73–3.64 (m, [4 H], N=C*H*_2_CO_2_), 4.14–3.95 (d, [1 H], ^2^*J*_HH_ 14.0 Hz, Bz CH_2_), 4.70–4.46 (d, [1 H], ^2^*J*_HH_ 13.9 Hz, Bz CH_2_), 7.45 ppm (m, [5 H], Ar CH); ^19^F{^1^H} NMR (300 MHz, CD_3_OD, 298 K): *δ*=−184.6 ppm (br s); ESI^+^ (MeOH): *m*/*z*=402.1 (100 %) [GaL]^+^; IR (Nujol): $\tilde \nu $
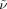
=3420 (H_2_O), 1665, 1650 (C=O), 568 cm^−1^ ($\tilde \nu $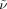 Ga=F). Slow evaporation of the reaction solution gave large yellow crystals suitable for single-crystal X-ray diffraction analysis.

*Method 2:* A solution of KF (0.006 g, 0.095 mmol) in H_2_O (3 mL) was added dropwise to a solution of [GaCl(L)] (0.040 g, 0.095 mmol) in H_2_O (3 mL). The yellow solution was stirred at RT for 90 min. The volatiles were removed under high vacuum upon gentle heating (ca. 40 °C). The yellow solid was washed with MeOH. The solution was filtered to remove insoluble particulates. Removal of the volatiles in vacuo gave the product as a yellow solid. Yield 0.017 g, 60 %; spectroscopic data are as those reported for method 1.

*Method 3:* Ga(NO_3_)_3_⋅9 H_2_O (0.030 g, 0.072 mmol), H_2_L⋅HCl (0.024 g, 0.072 mmol) and KF (0.004 g, 0.072 mmol) were added to freshly distilled H_2_O (5 mL). The resulting yellow solution was stirred at RT for 90 min. The volatiles were removed under high vacuum upon gentle heating (ca. 40 °C). The yellow solid was washed with MeOH. The insoluble particulates were removed by filtration, and the solution was concentrated in vacuo to give the complex as a yellow solid. Yield: 0.016 g, 58 %; spectroscopic data are as those reported for method 1.

*Method 4*: As was described for method 3, but using Ga(NO_3_)_3_⋅9 H_2_O (0.025 g, 0.060 mmol), Li_2_L (0.021 g, 0.060 mmol) and KF (0.003 g, 0.060 mmol). Yield: 0.012 g, 44 % (yellow solid.); spectroscopic data are as those reported for method 1.

*Method 5:* A solution of KF (0.005 g, 0.086 mmol) in H_2_O (1 mL) and Li_2_L (0.033 g, 0.086 mmol) in H_2_O (5 mL) was added simultaneously to powdered GaCl_3_ (0.015 g, 0.086 mmol). Addition of the aqueous solutions resulted in an exothermic reaction and the formation of an orange solution. The mixture was stirred at RT for 90 min, over which time the solution darkened. The volatiles were removed under high vacuum upon gentle heating (ca. 40 °C). The yellow orange solid was washed with MeOH. The insoluble particulates were removed by filtration, and the solution was concentrated in vacuo to give the desired complex as a yellow solid. Yield: 0.026 g, 50 %; spectroscopic data are as those reported for method 1.

### Radiolabelling experiments

Experiments were analysed on a Gilson 322 HPLC system with a Gilson 156 UV detector. Dionex chromeleon 6.8 chromatography data-recording software (Thermo-Fisher, UK) was used to integrate the UV and radiochemical peak areas. Analytical HPLC: Luna 5μ C18(2) 250×4.6 mm (mobile phase A=10 mm ammonium acetate, B=100 % MeCN); flow rate 1 mL min^−1^; gradient 0–15 min (10–90 % B), 15–20 min (90 % B), 20–21 min (90–10 % B), 21–26.5 min (10 % B). Product purification was accomplished by using a Waters HLB SPE cartridge (WAT0942260) pre-conditioned with EtOH (5 mL) and H_2_O (10 mL).

ESI^+^ mass spectra were recorded from direct injection of the products onto a Thermo Finnigan mass spectrometer fitted with an LCQ advantage MS pump.

NaOAc buffer solutions were prepared by combination of the appropriate volumes of 2 mm NaOAc and 2 mm HOAc.

### ^18^F Radiolabelling of [GaCl(L)]⋅2 H_2_O

[GaCl(L)]⋅2 H_2_O (0.1 mg, 210 nmol) was dissolved in NaOAc buffer (0.4 mL; pH 4) and added to ^18^F (500–1000 MBq) in NaOAc buffer (0.1 mL; pH 4). The solution was stirred rapidly for 30 min at 80 °C. A sample of the reaction mixture (100 μL) was taken, and made up to 1 mL in H_2_O. This solution (100 μL) was injected onto an RP HPLC system. Peak 1: *R*_t_=2.5 min (^18^F^−^) 30–35 %, 5.9–6.5 min (product) 65–70 % incorporation.

### HLB Purification of [Ga^18^F(L)]

The crude reaction mixture was diluted into NaOAc buffer (10 mL), loaded onto a pre-conditioned HLB cartridge and washed with H_2_O (3×1 mL). The product was eluted with EtOH/H_2_O (2×0.2 mL 1:1). Cartridge purification gave approximately 50 % yield of desired compound in up to 100 MBq mL^−1^ radioactive concentration.

### Stability studies

The HLB-purified product was formulated into a number of solvent compositions of various pH so that the total formulated volume was 1 mL. Aliquots (100 μL) of the formulated product were taken and diluted further prior to injection on to the analytical HPLC.

### In situ one-pot labelling reaction to form [Ga^18^F(L)]

Ga(NO_3_)_3_⋅9 H_2_O (1 mg in 0.5 mL NaOAc (pH 4), 2.40 μmol) and Li_2_L (0.5 mg in 0.5 mL NaOAc (pH 4), 2.40 nmol) were added to ^18^F/H_2_^18^O (300–500 MBq). The mixture was heated to 80 °C for 30 min upon vigorous stirring. The solution was cooled to RT. A sample of the reaction mixture (100 μL) was removed and made up to 1 mL in NaOAc. This solution (100 μL) was injected onto an RP HPLC system. Peak 1: *R*_t_=2.5 min (^18^F^−^) 10 %, 3.1 min (unidentified F^−^ salt) 10 %, 6.0 min (product) 80 %. The crude reaction mixture was purified by HLB cartridge purification.

### X-Ray crystallography

Crystals were obtained as described above. Details of the crystallographic data collection and refinement are in Table 1. Rigaku AFC12 goniometer equipped with an enhanced sensitivity (HG) Saturn724+ detector mounted at the window of an FR-E+ SuperBright molybdenum rotating anode generator (*λ*_1_=0.71073 Å) with VHF Varimax optics (70 μm focus). Cell determination, data collection, data reduction, cell refinement and absorption correction: CrystalClear-SM Expert 2.0 r7.[16] Structure solution and refinement were routine by using WinGX and software packages within,[17] except for [GaCl(L)]⋅2 H_2_O (see the Supporting Information) for which only very small, weakly diffracting crystals were obtained despite numerous recrystallization attempts. This species is isostructural with [GaF(L)]⋅2 H_2_O and also with [AlF(L)]⋅2 H_2_O.[5a] CCDC-1030903 http://www.ccdc.cam.ac.uk/cgi-bin/catreq.cgi([GaCl(L)]⋅MeCN), CCDC-1024100 http://www.ccdc.cam.ac.uk/cgi-bin/catreq.cgi([GaF(L)]⋅2 H_2_O), and CCDC-1024101 http://www.ccdc.cam.ac.uk/cgi-bin/catreq.cgi([GaCl(L)]⋅2 H_2_O) contain the supplementary crystallographic data for this paper. These data can be obtained free of charge from The Cambridge Crystallographic Data Centre via http://www.ccdc.cam.ac.uk/data_request/cif.

**Table 1:** Selected crystallographic data.

Complex	[GaCl(L)]⋅MeCN	[GaF(L)]⋅2 H_2_O
formula	C_19_H_26_ClGaN_4_O_4_	C_17_H_27_FGaN_3_O_6_
*M* [g^−1^ mol^−1^]	479.61	458.14
*T* [K]	100(2)	100(2)
crystal system	monoclinic	monoclinic
space group (no.)	*P*2_1_/*n* (14)	*P*2_1_/*c* (14)
*a* [Å]	12.018(2)	19.883(3)
*b* [Å]	13.032(2)	7.1449(7)
*c* [Å]	13.230(2)	13.8880(15)
*α* [°]	90	90
*β* [°]	102.373(2)	106.465(8)
*γ* [°]	90	90
*U* [Å^3^]	2024.0(6)	1892.1(4)
*Z*	4	4
*μ* (Mo_K*α*_) [mm^−1^]	1.526	1.505
*F*(000)	992	952
total reflections	10 274	12 106
unique reflections	4626	4303
*R*_int_	0.029	0.089
*R*_1_ [*I*_o_>2*σ*(*I*_o_)]	0.030	0.067
*R*_1_ (all data)	0.037	0.0907
*wR*_2_ [*I_o_*>2*σ*(*I_o_*)]	0.077	0.144
*wR*_2_ (all data)	0.080	0.155

## References

[1a] Hull KL, Anani WQ, Sanford MS (2006). J. Am. Chem. Soc.

[1b] Lee E, Kamlet AS, Powers DC, Neumann CN, Boursalian GB, Furuya T, Choi DC, Hooker JM, Ritter T (2011). Science.

[1c] Furuya T, Kamlet AS, Ritter T (2011). Nature.

[1d] Tang PP, Furuya T, Ritter T (2010). J. Am. Chem. Soc.

[1e] Liu W, Huang X, Cheng M-J, Nielsen RJ, Goddard WA, Groves JT (2012). Science.

[1f] Watson DA, Su M, Teverovskiy G, Zhang Y, Garc’ıa-Fortanet J, Kinzel T, Buchwald SL (2009). Science.

[2a] Ting R, Adam MJ, Ruth TJ, Perrin DM (2005). J. Am. Chem. Soc.

[2b] Ting R, Harwig C, auf dem Keller U, McCormick S, Austin P, Overall CM, Adam MJ, Ruth TJ, Perrin DM (2008). J. Am. Chem. Soc.

[2c] Li Z, Chansaenpak K, Liu S, Wade CR, Conti PS, Gabbäı FP (2012). Med.Chem. Commun.

[2d] Ting R, Aguilera TA, Crisp JL, Hall DJ, Eckelman WC, Vera DR, Tsien RY (2010). Bioconjugate Chem.

[3] For examples see

[3a] Schirrmacher R, Bradtmöller G, Schirrmacher E, Thews O, Tillmanns J, Siessmeier T, Buchholz HG, Bartenstein P, Wängler B, Niemeyer CM, Jurkschat K (2006). Angew. Chem. Int. Ed.

[3b] Wängler B, Kostikov AP, Niedermoser S, Chin J, Orchowski K, Schirrmacher E, Iovkova-Berends L, Jurkschat K, Wängler C, Schirrmacher R (2012). Nat. Protoc.

[3c] Wängler C, Waser B, Alke A, Iovkova L, Buchholz HG, Niedermoser S, Jurkschat K, Fottner C, Bartenstein P, Schirrmacher R, Reubi JC, Wester HJ, Wängler B (2010). Bioconjugate Chem.

[3d] Wängler C, Niedermoser S, Chin J, Orchowski K, Schirrmacher E, Jurkschat K, Iovkova-Berends L, Kostikov AP, Schirrmacher R, Wängler B (2012). Nat. Protoc.

[4a] Laverman P, McBride W, Sharkey R, Eek A, Joosten L, Oyen W, Goldenberg D, Boerman O (2010). J. Nucl. Med.

[4b] McBride W, D′Souza C, Sharkey R, Karacay H, Rossi E, Chang C, Goldenberg D (2010). Bioconjugate Chem.

[4c] McBride W, Sharkey R, Karacay H, D′Souza C, Rosso E, Laverman P, Chang C, Boerman O, Goldenberg D (2009). J. Nucl. Med.

[4d] McBride W, D′Souza C, Karacay H, Sharkey R, Goldenberg D (2012). Bioconjugate Chem.

[4e] D′Souza CA, McBride WJ, Sharkey RM, Todaro LJ, Goldenberg DM (2011). Bioconjugate Chem.

[4f] Lütje S, Franssen GM, Sharkey RM, Laverman P, Rossi EA, Goldenberg DM, Oyen WJG, Boerman OC, McBride WJ (2014). Bioconjugate Chem.

[4g] McBride WJ, Goldenberg DM, Sharkey RM (2014). J. Nucl. Med.

[5a] Shetty D, Choi SY, Jeong JM, Lee JY, Hoigebazar L, Lee Y-S, Lee DDS, Chung JK, Lee MC, Chung YK (2011). Chem. Commun.

[5b] Glaser M, Iveson P, Hoppmann S, Indrevoll B, Wilson A, Arukwe J, Danikas A, Bhalla R, Hiscock D (2013). J. Nucl. Med.

[6] Bhalla R, Darby C, Levason W, Luthra SK, McRobbie G, Reid G, Sanderson G, Zhang W (2014). Chem. Sci.

[7a] Aldridge S, Downs AJ (2011). The Group 13 Metals Aluminium, Gallium, Indium and Thallium: Chemical Patterns and Peculiarities.

[7b] Bandoli G, Dolmella A, Tisato G, Porchia M, Refosco F (2009). Coord. Chem. Rev.

[8] Wan W, Guo N, Pan D, Yu C, Wenig Y, Luo S, Ding H, Xu Y, Wang L, Lang L, Xie Q, Yang M, Chen X (2013). J. Nucl. Med.

[9a] Kimura S, Bill E, Bothe E, Weyhermüller T, Wieghardt K (2001). J. Am. Chem. Soc.

[9b] Song Y-F, Berry JF, Bill E, Bothe E, Weyhermüller T, Wieghardt K (2007). Inorg. Chem.

[10] Carty AJ, Tuck DG (1975). Prog. Inorg. Chem.

[11] Timoshkin AY, Bodensteiner M, Sevastianova TN, Lisovenko AS, Davydova EI, Scheer M, Grassl C, Butlak AV (2012). Inorg. Chem.

[12] Kazakov IV, Bodensteiner M, Timoshkin AY (2014). Acta Crystallogr. C.

[13] Cordero B, Gómez V, Platero-Prats AE, Revés M, Echeverría J, Cremades E, Barragán F, Alvarez S (2008). Dalton Trans.

[14] Bodor A, Tóth I, Bànyai I, Szabò Z, Hefter GT (2000). Inorg. Chem.

[15] Burt J, Levason W, Light ME, Reid G (2014). Dalton Trans.

[16] Rigaku Corporation, Tokyo, Japan, **2011**

[17] Farrugia LJ (2012). J. Appl. Crystallogr.

