# Supplementary material for: Radiofluorination of a Pre-formed Gallium(III) Aza-macrocyclic Complex: Towards Next-Generation Positron Emission Tomography (PET) Imaging Agents
Source: Chemistry. 2015 Feb 4;21(12):4688–94. doi: 10.1002/chem.201405812 (PMC4515101; doi:10.1002/chem.201405812)
Supplement: Supplementary file 1 [file chem0021-4688-sd1.pdf]

CHEMISTRY
A European Journal

Supporting Information

Radiofluorination of a Pre-formed Gallium(III) Aza-macrocyclic Complex: Towards Next-Generation Positron Emission Tomography (PET) Imaging Agents

Rajiv Bhalla,[a] William Levason,[b] Sajinder K. Luthra,[c] Graeme McRobbie,[c] George Sanderson,[b] and Gillian Reid*[b]

chem_201405812_sm_miscellaneous_information.pdf

Supporting Information

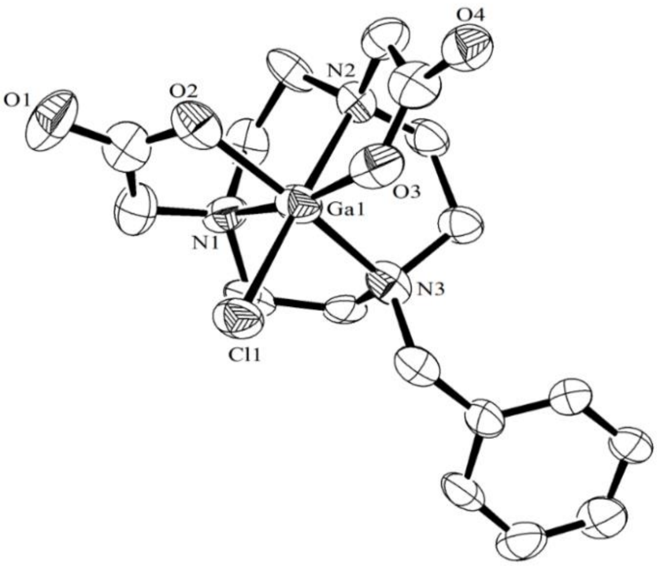

(a)

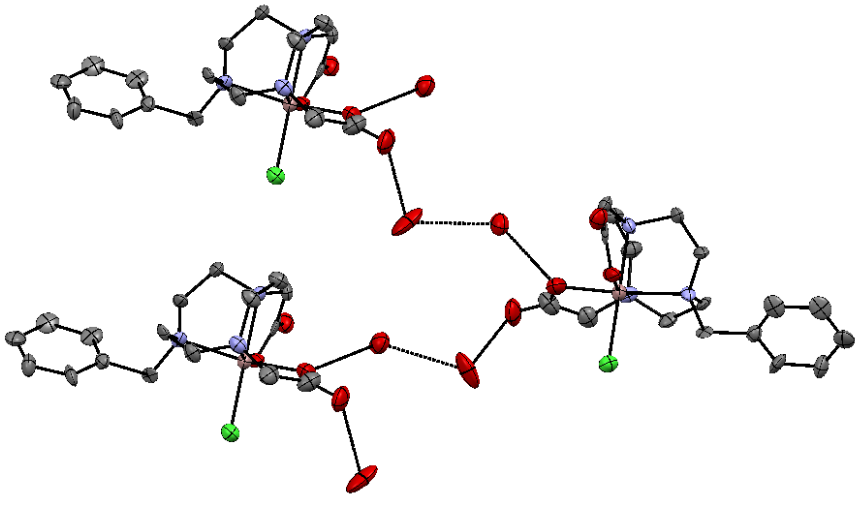

(b)

Figure S1 (a) ORTEP representation of the gallium species in $[GaCl(L)]\cdot 2H_2O$ with atom numbering scheme. H atoms are omitted for clarity (the H atoms associated with the lattice H_2O molecules were not located in the difference map). Thermal ellipsoids are drawn at 50% probability. Selected bond lengths (Å): Ga1−Cl1 2.266(4), Ga1−O2 1.888(13), Ga1−O4 2.010(11), Ga1−N1 2.122(11), Ga1−N2 2.094(13), Ga1−N3 2.147(13); (b) Diagram showing a portion of the extended H-bonded structure of $[GaCl(L)]\cdot 2H_2O$ (Ga = pink; Cl = green; O = red; N = blue; C = grey).

Table S1. Selected crystallographic data

Complex	$[GaCl(L)]\cdot 2H_2O$
Formula	$C_{17}H_{27}ClGaN_3O_6$
M / g mol^{-1}	474.59
Temp. / K	100(2)
Crystal system	Monoclinic
Space group (no.)	$P2_1/c$ (14)
a / Å	18.648(7)
b / Å	8.132(3)
c / Å	13.197(5)
α / º	90
β / º	101.328(12)
γ / º	90
U / Å³	1962.2(13)
Z	4
μ(Mo-K_{α}) mm^{-1}	1.579
F(000)	984
Total reflections	9388
Unique reflections	4438
R_{int}	0.221
R_1 [$I_o > 2\sigma(I_o)$]	0.141
R_1 (all data)	0.293
wR_2 [$I_o > 2\sigma(I_o)$]	0.322
wR_2 (all data)	0.412

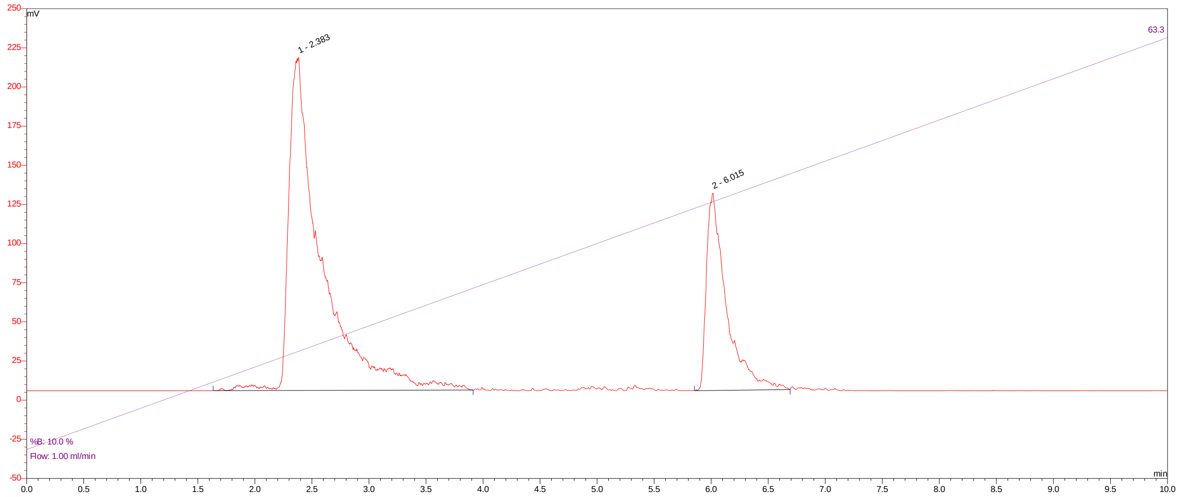

Figure S2: HPLC radio trace of the crude reaction mixture from the reaction of [GaCl(L)]·2H_2O with $^{18}F/^{18}OH_2$ in NaOAC (pH 4), stirred at room temperature for 30 mins. Rt 2.4 mins (70%) $^{18}F^{-}$, Rt 6.0 mins (27%) [Ga^{18}F(L)].

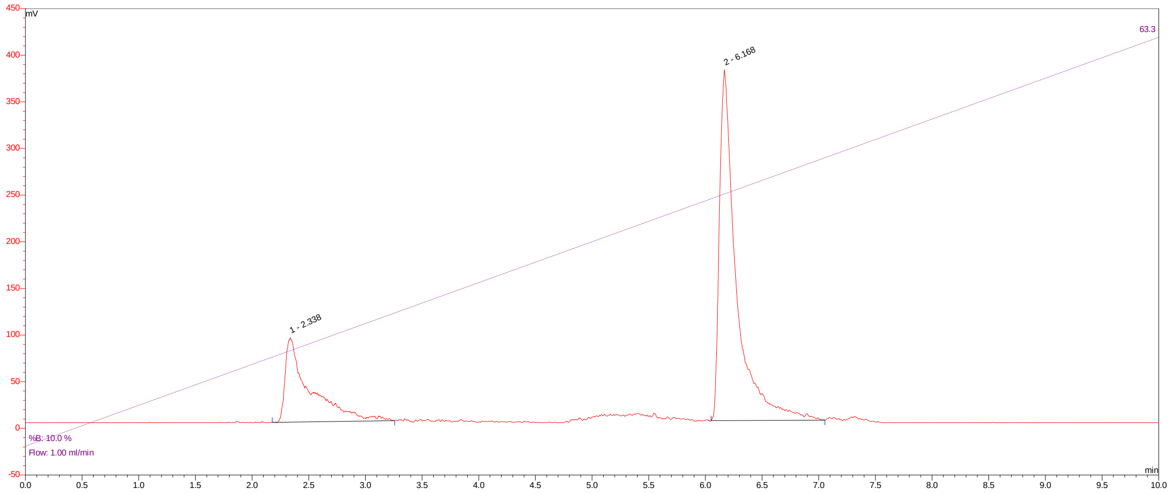

Figure S3: HPLC radio trace of the crude reaction mixture from the reaction of [GaCl(L)]·2H_2O with $^{18}F/^{18}OH_2$ in NaOAC (pH 4), heated to 80°C for 30 mins. Rt 2.3 mins (30%) $^{18}F^{-}$, Rt 6.2 mins (70%) [Ga^{18}F(L)].

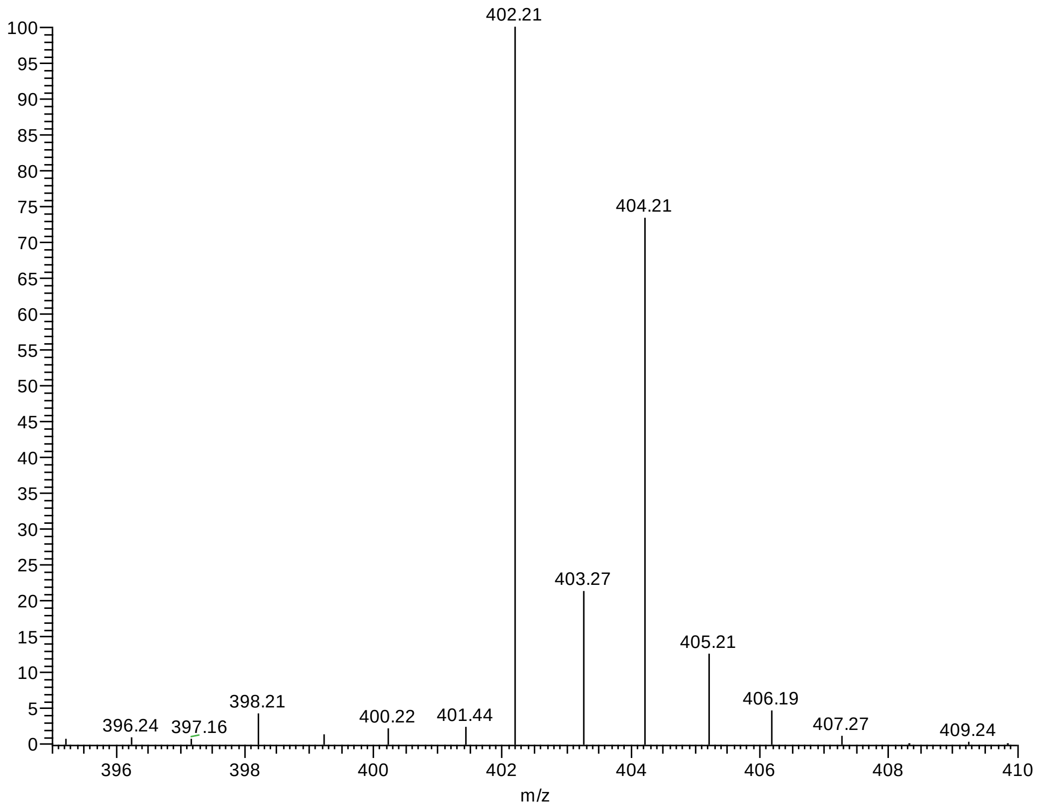

Figure S4: ESI$^+$ mass spectrum and isotope pattern for $[Ga(L)]^+$ *m/z* = 402.2 (100%).

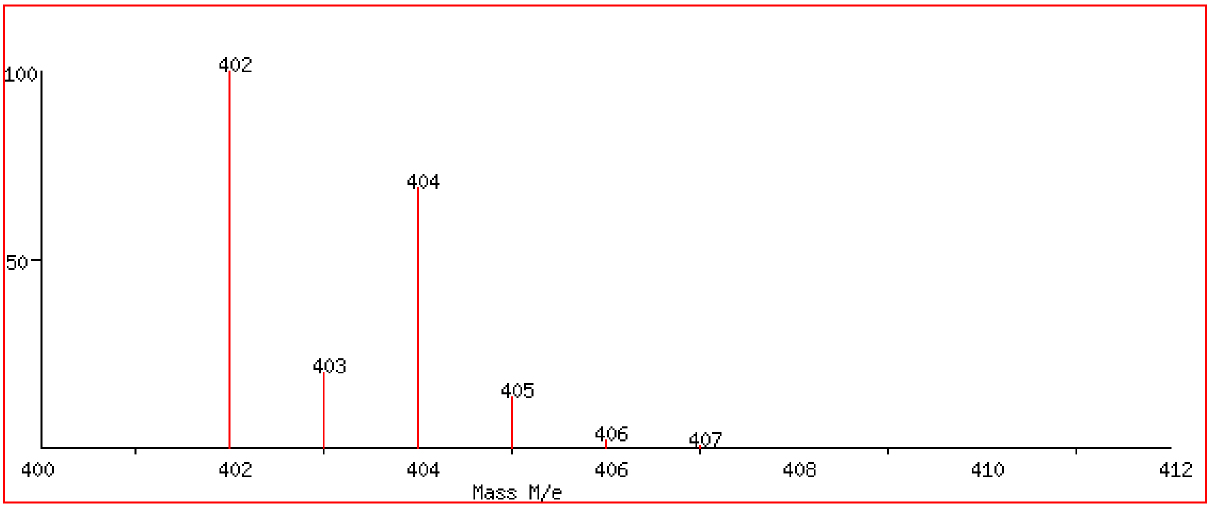

Figure S5: Predicted peak position and isotope pattern for the species $[Ga(L)]^+$.

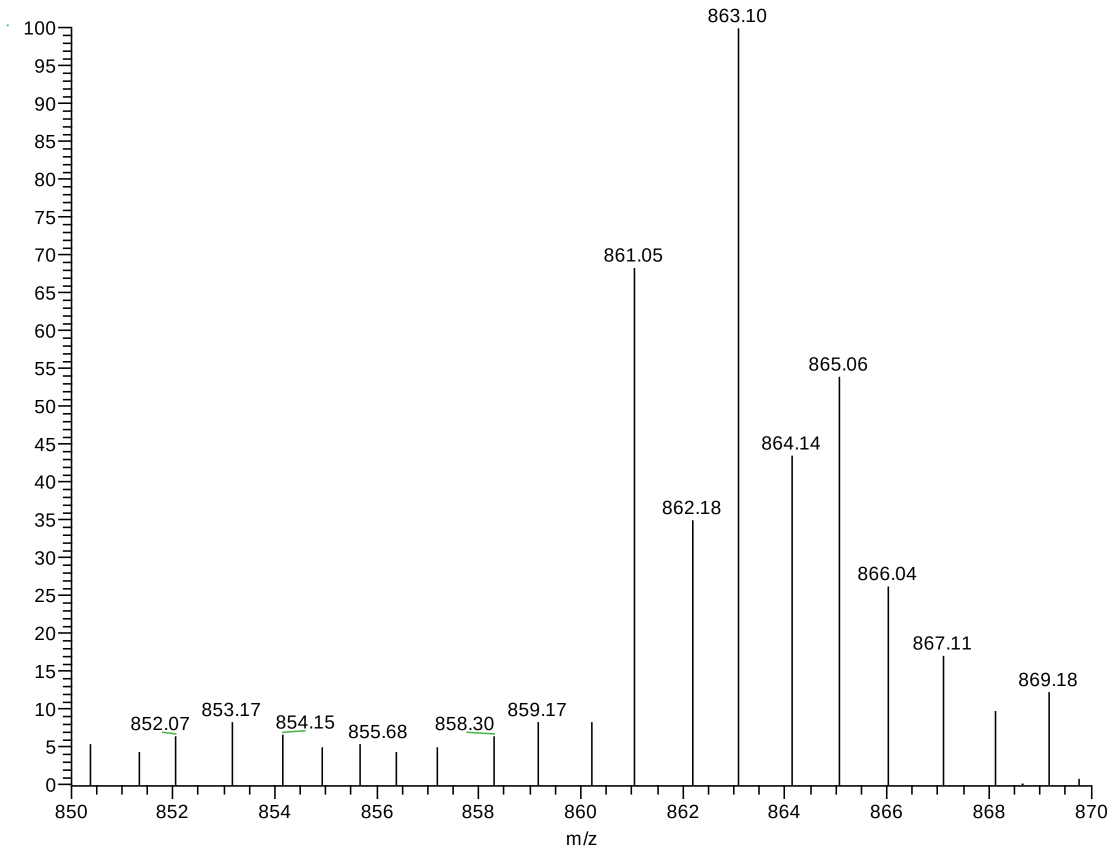

Figure S6: ESI$^+$ mass spectrum and isotope pattern for $[\{GaF(L)\}_2{+}H_3O]^+$ *m/z* 863.1 (100%).

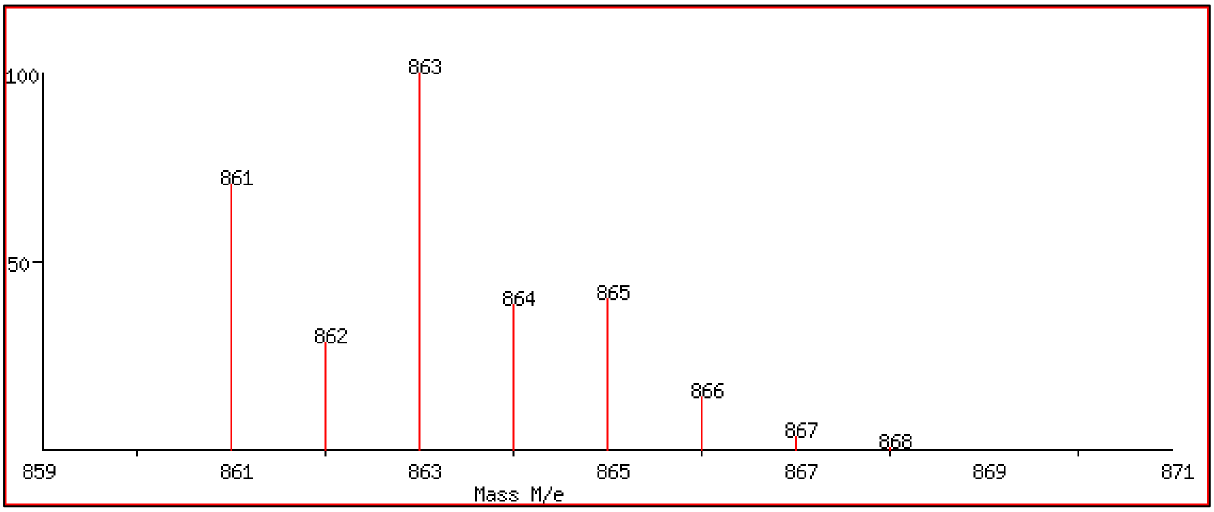

Figure S7: Predicted peak position and isotope pattern for the species $[\{GaF(L)\}_2{+}H_3O]^+$.

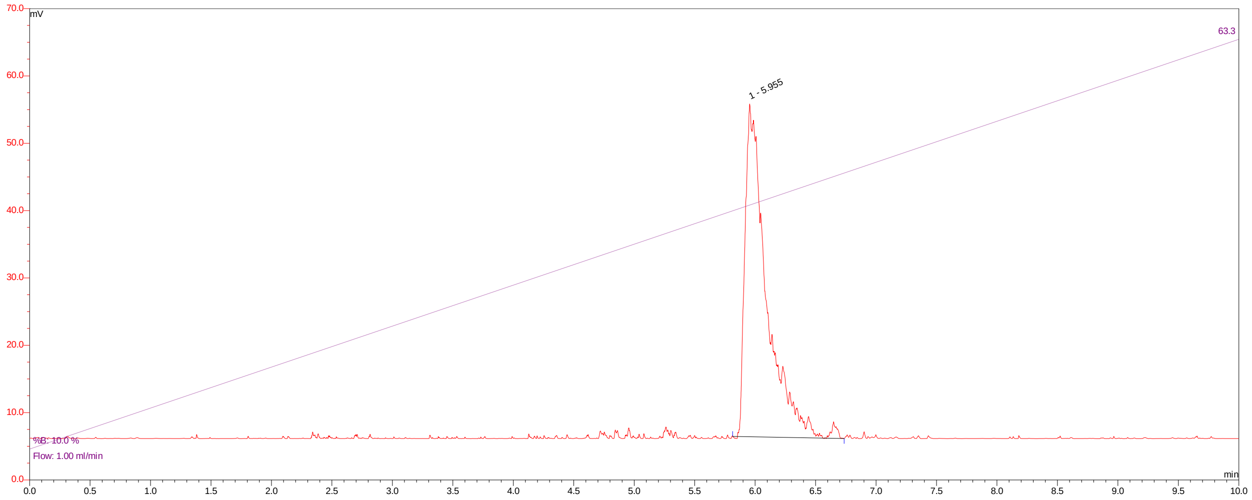

Figure S8a: HPLC radio trace of the HLB purified product formulated in 10% EtOH/NaOAc (pH 6) at t = 20 mins. Rt 6.0 mins (99%) [$Ga^{18}F(L)$].

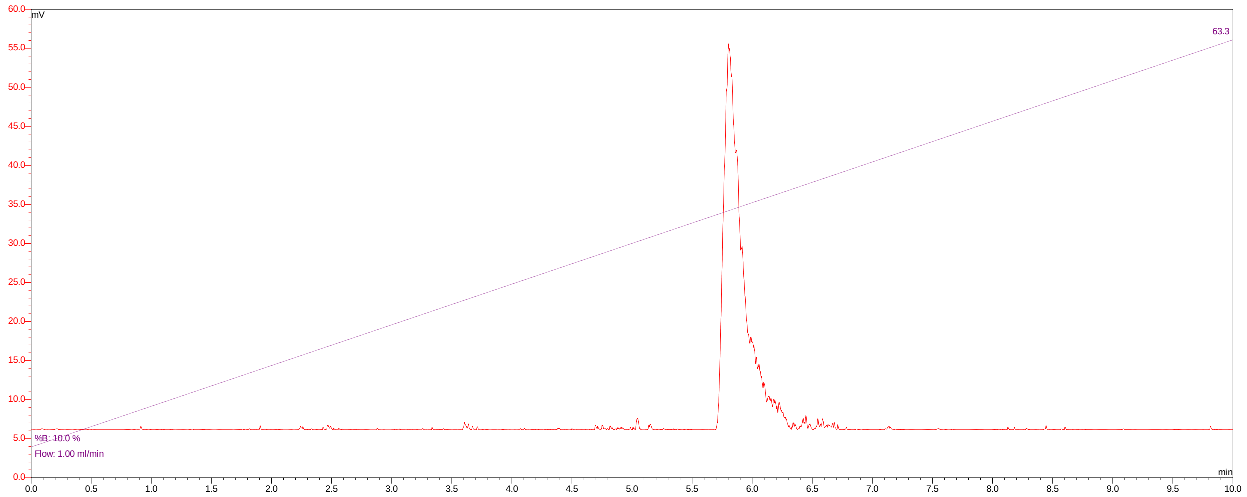

Figure S8b: HPLC radio trace of the HLB purified product formulated in 10% EtOH/NaOAc (pH 6) at t = 45 mins. Rt 5.9 mins (99%) [$Ga^{18}F(L)$].

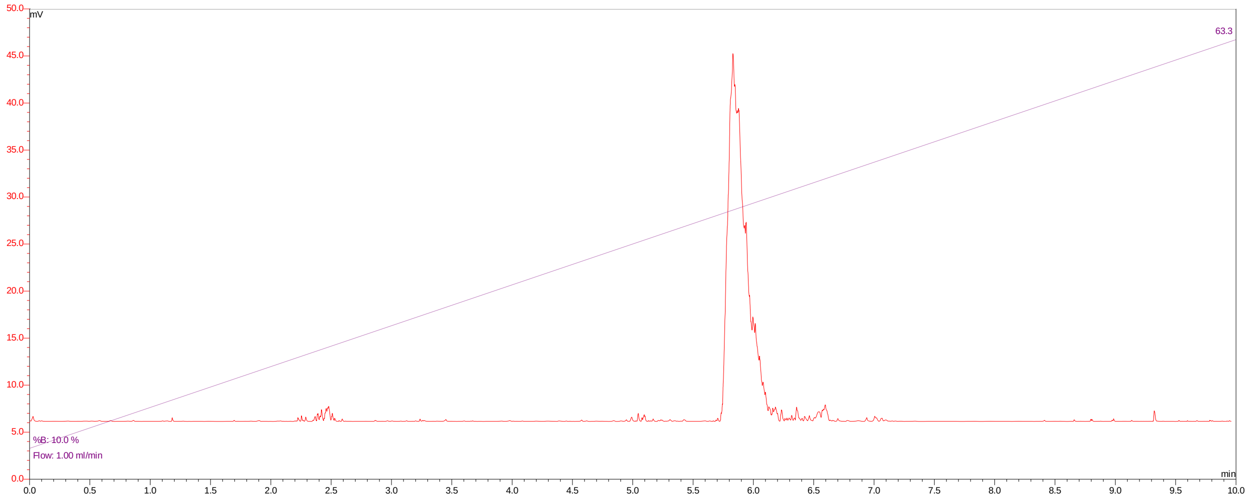

Figure S8c: HPLC radio trace of the HLB purified product formulated in 10% EtOH/NaOAc (pH 6) at t = 90 mins. Rt 5.9 mins (96%) [$Ga^{18}F(L)$].

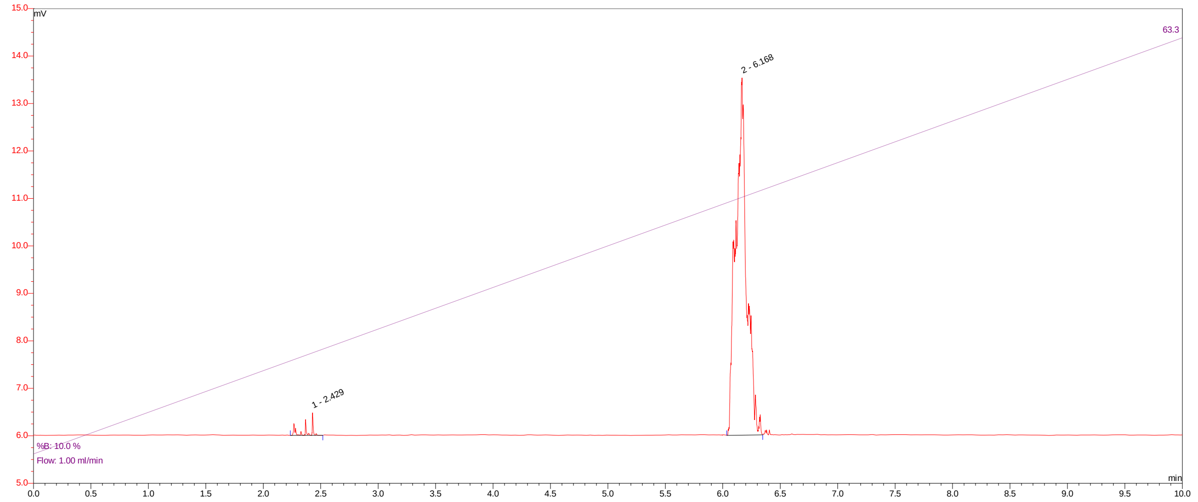

Figure S9a: HPLC radio trace of the HLB purified product formulated in 10% EtOH/PBS (pH 7.5) at t = ‘zero’. Rt 2.4 mins (2%) $^{18}F^{-}$, Rt 6.2 mins (98%) [Ga^{18}F(L)].

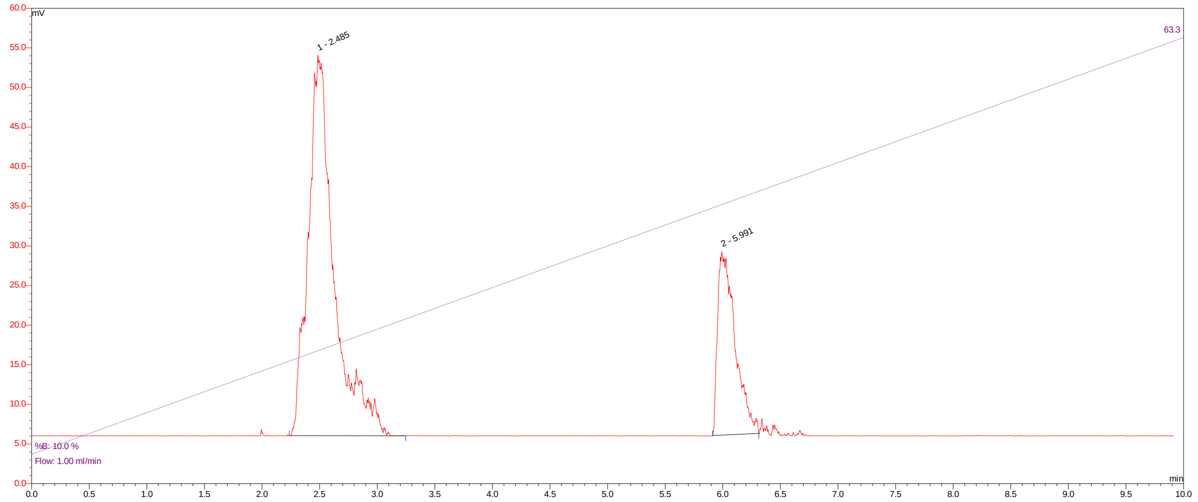

Figure S9b: HPLC radio trace of the HLB purified product formulated in 10% EtOH/PBS (pH 7.5) at t = 45 mins. Rt 2.5 mins (55%) $^{18}F^{-}$, Rt 6.0 mins (45%) [Ga^{18}F(L)].

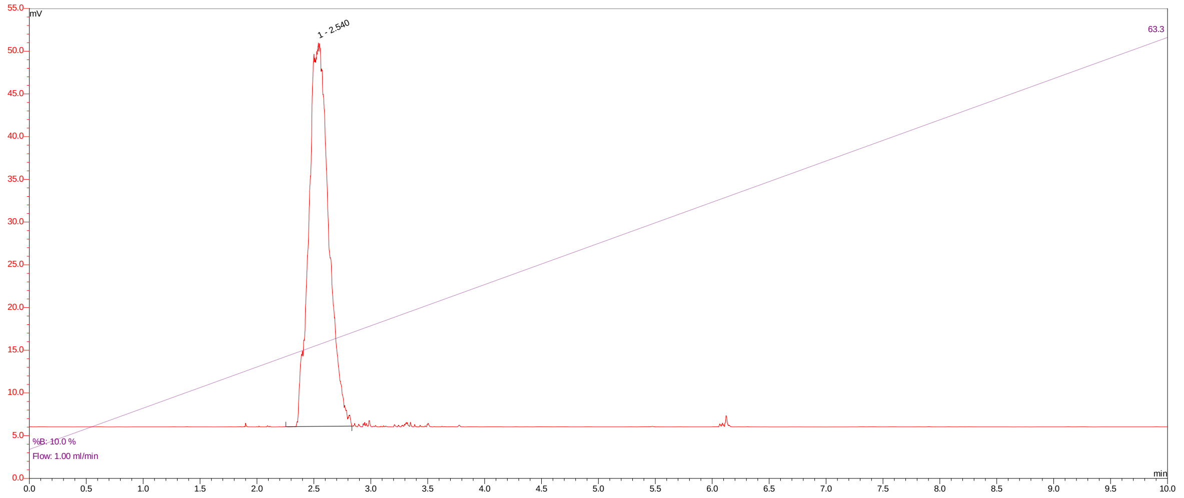

Figure S9c: HPLC radio trace of the HLB purified product formulated in 10% EtOH/PBS (pH 7.4) at t = 120 mins. Rt 2.5 mins (100%) $^{18}F^{-}$.

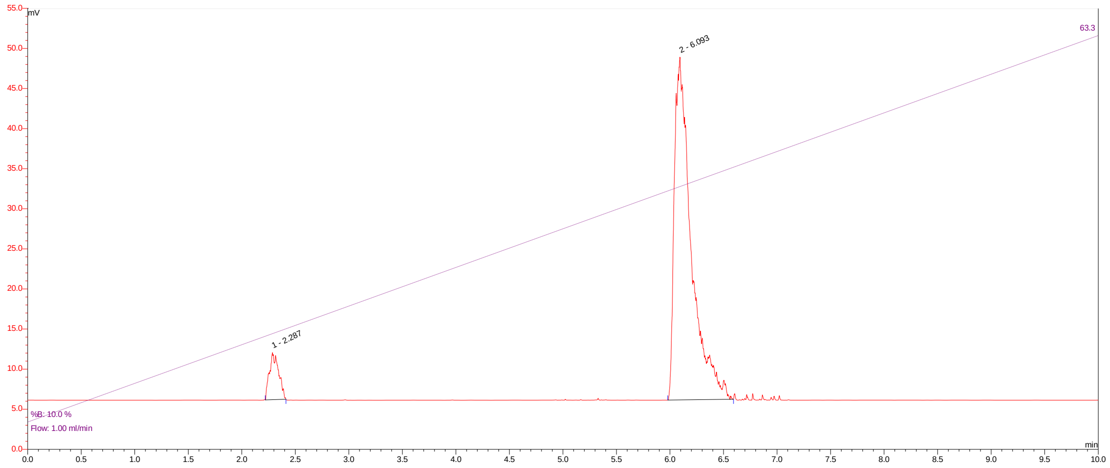

Figure S10a: HPLC radio trace of the HLB purified product formulated in 10% EtOH/HSA (pH 7.5) at t = ‘zero’. Rt 2.3 mins (7%) $^{18}F^{-}$, Rt 6.2 mins (93%) [Ga^{18}F(L)].

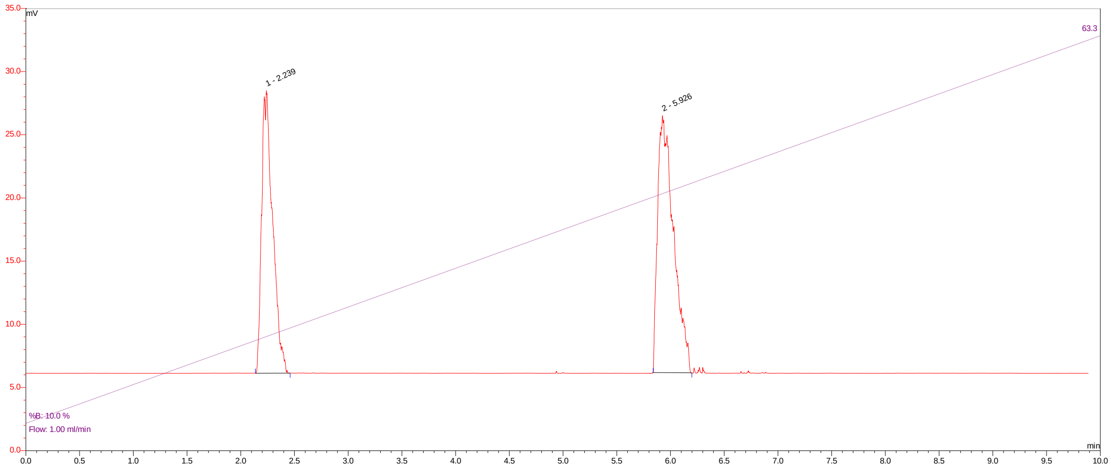

Figure S10b: HPLC radio trace of the HLB purified product formulated in 10% EtOH/HSA (pH 7.5) at t = 30 mins. Rt 2.2 mins (43%) $^{18}F^{-}$, Rt 6.2 mins (57%) [Ga^{18}F(L)].

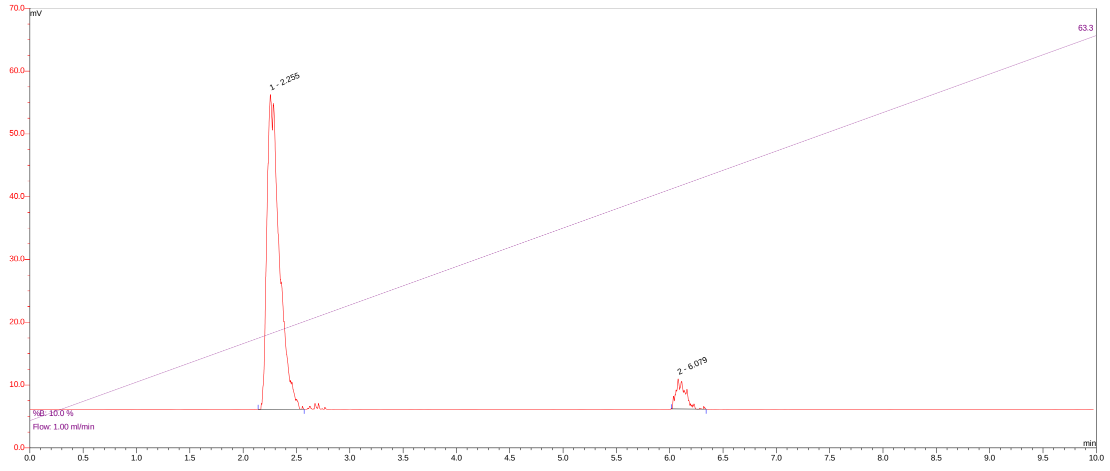

Figure S10c: HPLC radio trace of the HLB purified product formulated in 10% EtOH/HSA (pH 7.5) at t = 90 mins. Rt 2.3 mins (93%) $^{18}F^{-}$, Rt 6.1 mins (7%) [Ga^{18}F(L)].

Table S2 % RCP of specific formulations as a function of time

Formulation (pH)#	RCP/% (time/ mins.)*			
EtOH/PBS (7.5)	98 (0)	40 (30)	25 (45)	2 (120)
HSA (7.4)	93 (0)	57 (30)	7 (90)	
EtOH/H_2O (7)	91 (0)	76 (30)	34 (120)	0 (180)
EtOH/10mM NH_4OAc (7)	90 (0)	49 (30)	9 (45)	4 (90)
EtOH/NaOAc (6)	99 (20)	99 (45)	96 (90)	-
EtOH/NaOAc (5)	99 (45)	99 (90)	99 (180)	-
EtOH/NaOAc (4)	99 (0)	99 (90)	99 (120)	99 (240)

# EtOH volume was approximately 10% of total formulation volume (e.g. 0.1 mL EtOH in 1 mL formulated product).
* RCP estimated from integration of radiochemical peaks in HPLC chromatographs.
